# University students’ career adaptability as a mediator between cognitive emotion regulation and career decision-making self-efficacy

**DOI:** 10.3389/fpsyg.2022.896492

**Published:** 2022-10-05

**Authors:** Ahram Lee, Eunju Jung

**Affiliations:** ^1^Department of Education, Sejong University, Seoul, South Korea; ^2^Graduate School of Education, Sejong University, Seoul, South Korea

**Keywords:** career construction model of adaptation, cognitive emotion regulation, career adaptability, career decision-making self-efficacy, university students

## Abstract

As modern society experiences rapid changes, the unpredictability of the labor market is increasing. University students preparing to join the workforce may experience increased anxiety and stress due to the heightened uncertainty regarding their career plans. Regulating such negative emotions and adjusting to the changing circumstances may influence their career development. Thus, the current study aimed to investigate the relationship between cognitive emotion regulation (CER) — specifically adaptive CER and maladaptive CER — and career decision-making self-efficacy (CDMSE), with career adaptability (CA) as a mediating factor. The path analysis model consisting of adaptive CER, maladaptive CER, CA, and CDMSE was tested with 357 Korean university students who were facing the school-to-work transition. The results of the study were as follows. First, adaptive CER was positively related to CA and CDMSE, while maladaptive CER was negatively related to CA only. Second, CA and CDMSE were positively related. Third, CA partially mediated the relationship between adaptive CER and CDMSE and fully mediated the relationship between maladaptive CER and CDMSE. Based on these results, theoretical and practical implications are proposed, and the limitations of the study are discussed.

## Introduction

Modern society is changing rapidly and becoming more complex, and various factors such as the great recession around the world, increased inequalities caused by polarized jobs, and the advancement of technology, including automation and artificial intelligence, are affecting the labor market (Blustein, 2019). The prolonged global impact of COVID-19 is also a factor that aggravates uncertainty in the world of work, increasing the risk of layoffs, reducing new job opportunities, and leading to employment disparities (Autin et al., 2020; Restubog et al., 2020). In the winter of 2020, when the current study was conducted, COVID-19 was at its peak in South Korea, and dismal news about the job market was frequently heard. For instance, according to the Korean Statistical Information Service (2021), the employment rate in 2020 for those aged 20–29 fell 2.5 percentage points compared to that in 2019, while corporations have been postponing their recruitment processes.

Due to such various changes, the labor market is becoming increasingly unpredictable as new opportunities arise and traditional jobs disappear. In such fluid and unstable times, university students who are preparing to join the workforce can no longer expect a linear development in their careers (Pyo and Yang, 2020). They should not only focus on gaining domain-specific knowledge and skills but also on fostering resources that can be applied flexibly in various areas. To assist university students facing the school-to-work transition, it is necessary to identify factors that relate to coping with the volatile circumstances of today’s world.

Recent studies have focused on career adaptability (CA) as an important factor that needs to be enhanced in times of unpredictability, especially during the pandemic (Wen et al., 2020; Lau et al., 2021). CA, one of the key components of Savickas (2013) career construction model of adaptation, refers to individuals’ psychosocial resources that are necessary for coping with the insecure career landscape of modern society (Savickas, 2013; Rudolph et al., 2017; Johnston, 2018). Career construction theory understands career development from a contextual perspective, emphasizing individuals’ interaction with and adaptation to the ever-changing environment, and CA can help individuals construct their careers through managing unfamiliar tasks and transitions (Savickas, 2013). Thus, investigating university students’ CA has important value because CA not only helps students successfully transition to employment (Koen et al., 2012), but also is significantly related to positive variables such as higher employment quality (Koen et al., 2012), career satisfaction (Chan and Mai, 2015), general and professional wellbeing (Maggiori et al., 2013), as well as self-regulation, career construction, and academic engagement (Merino-Tejedor et al., 2016).

Along with empirical studies highlighting the role of CA, there have been efforts to verify the career construction model of adaptation which illustrates the mechanism by which CA operates in the process of adaptation (Savickas and Porfeli, 2012; Hirschi et al., 2015; Johnston, 2018). The model of adaptation explains the notion, to adapt, in a sequence: *adaptivity* or adaptive readiness refers to a rather stable personality trait of flexibility and willingness to adapt; *adaptability* indicates the psychosocial resources individuals have to cope with various career-related challenges and tasks; *adapting* or adaptive responses refers to behaviors that function to accomplish developmental tasks in the changing context; and *adaptation* is the goodness of fit resulting from adaptive readiness, resources, and responses (Savickas and Porfeli, 2012; Savickas, 2013). Previous studies have examined variables that correspond to each of the sequential states (Hirschi et al., 2015; Rudolph et al., 2017; Johnston, 2018), but Shin and Lee (2018) suggested that there is a need to identify and verify additional variables that conform to the model in different contexts. Hence, the current study intends to find supporting evidence for the career construction model of adaptation in the Korean context.

To contribute to assisting university students in Korea who are in their school-to-work transition period, the current study examines CA in relation to career decision-making self-efficacy (CDMSE), which has been addressed as one of the adaptive responses in the career construction model of adaptation (Rudolph et al., 2017; Johnston, 2018). CDMSE refers to individuals’ beliefs about their ability to successfully perform tasks associated with career-related decision-making (Betz and Luzzo, 1996). A feeling of self-efficacy allows individuals to choose their career path and adapt in the transition period (Savickas, 2013); it also has positive effects on job performance and persistence in a career (Betz et al., 1996). Since self-efficacy has been postulated to lead to behavioral changes (Bandura, 1977), university students’ CDMSE may be an important factor that predicts future career decision-making behaviors. Moreover, CDMSE and CA have been examined as associating variables in numerous studies (Hou et al., 2014; Pambudi et al., 2019; Hamzah et al., 2021), and in Shin and Lee’s (2018) study, CA was found to have a mediating role between regulatory focus and CDMSE, confirming the model of adaptation. To contribute to the research, the current study also adheres to the career construction model of adaptation, proposing that CA will predict CDMSE.

Using the career construction model of adaptation, the current study also focuses on cognitive emotion regulation (CER) as adaptive readiness to reflect the impact of students’ regulation of negative emotions on CA and CDMSE. The role of emotions in career development has long been emphasized, as they are closely related to work experiences and career-related choices (Kidd, 1998, 2004). Specifically, positive affect was found to have a positive relationship with CDMSE (Park et al., 2021), and high CA was found to be associated with lower levels of negative affect (Fiori et al., 2015). However, there are no studies to the authors’ knowledge that have directly investigated the influence of emotion regulation on CA and CDMSE. Hence, the current study examines the cognitive strategies individuals used to regulate their emotions and investigates their relationship to CA and CDMSE per the career construction model of adaptation.

In sum, the purpose of the current study is to find supporting evidence for the relationships among CER — specifically adaptive CER and maladaptive CER — CA, and CDMSE in the framework of the career construction model of adaptation using a path analysis model. Also, the mediating effect of CA between either adaptive or maladaptive CER and CDMSE is examined using mediation analysis. Examining the relationships among the variables can provide supporting evidence to confirm the model of adaptation in the Korean context as well as insight for counselors and educators to assist university students in a successful transition to the world of work in an era of uncertainty.

## Literature review and hypotheses

### Career construction model of adaptation

Savickas’ (2013) career construction theory posits that individuals construct their careers based on subjective criteria for success. This is unlike the traditional theories of career development which emphasize objective criteria for success, such as the concept of person-career fit in Parsonian theory. That is, career construction theory says that individuals construct their unique career paths by giving meaning to their experiences and career behaviors, and it emphasizes adaptability in the context of transitions (Savickas, 2013). Based on career construction theory, Savickas (2013) suggests the model of adaptation in which four elements, namely adaptivity, adaptability, adapting, and adaptation, play sequential roles.

Adaptivity refers to adaptive readiness and is defined as a rather stable and context-general personality trait of flexibility and willingness to adapt when faced with undefined career-related problems (Hirschi et al., 2015; Rudolph et al., 2017). Cognitive flexibility, proactivity, and the big five personality characteristics are some indicators of adaptivity (Savickas and Porfeli, 2012). Previous studies examining the career construction model of adaptation in the Korean context examined emotional and personality-related career decision-making difficulties (Park and Yoo, 2020) and regulatory focus (Shin and Lee, 2018) as adaptivity. The adaptivity factor used in the current study is CER which is an individual’s conscious way of coping with emotionally arousing situations (Garnefski and Kraaij, 2007). Emotion regulation is an individual’s coping strategy, and specific coping methods are closely related to personality traits (Vollrath and Torgersen, 2000; Connor-Smith and Flachsbart, 2007). Thus, the current study viewed CER as individual differences that function as adaptivity.

Adaptability is a psychosocial resource needed for individuals to manage various career-related challenges, tasks, and transitions, and it consists of four resources: concern, control, curiosity, and confidence (Savickas and Porfeli, 2012). Savickas and Porfeli (2012) developed the Career Adapt-Abilities Scale (CAAS) based on the career construction theory to measure these four dimensions. While they are theoretically distinguishable, they also have high correlations (Rudolph et al., 2017). Rudolph et al. (2017) suggested using a composite score rather than sub-scale scores for CA. Hence, the current study examines the total score of CA measured by CAAS.

Adapting refers to adaptive beliefs and behaviors that are implemented to accomplish career tasks in the changing context, and the adaptive responses include career planning, career exploration, career decision-making difficulties, and occupational self-efficacy (Hirschi et al., 2015). In the current study, CDMSE is considered an adaptive response in the career construction model of adaptation. CDMSE is an individual’s beliefs about their ability to successfully perform tasks related to career-related decision-making (Betz and Luzzo, 1996). The feeling of self-efficacy allows individuals to choose their own career path and adapt in the transition period (Savickas, 2013). CDMSE has been examined as an adaptive resource in previous studies as well (Duffy et al., 2015; Rudolph et al., 2017; Nilforooshan, 2020).

Adaptation is the final dimension in the career construction model of adaptation. It indicates the goodness of fit resulting from adaptive readiness, resources, and responses (Savickas and Porfeli, 2012; Savickas, 2013). Variables such as career identity, job stress, employability, and work performance are indicators of adaptation (Rudolph et al., 2017). The current study did not include an adaptation variable because the population being studied was university students who are in the preparation process and have yet to reach the final outcome of adaptation. However, as the model posits, adaptation is the final result of individuals’ adaptive beliefs and behaviors using various adaptive resources which draw upon personal readiness. Therefore, examining university students’ adaptivity, adaptability, and adapting may lead to predictions regarding their future adaptation.

### Cognitive emotion regulation and career decision-making self-efficacy

Cognitive emotion regulation is examined as adaptivity because emotions play a critical role in career development (Kidd, 1998, 2004). Also, in the current age of uncertainty, especially during the pandemic, regulating negative emotions has been addressed as an important factor for career readiness (Restubog et al., 2020). Emotional factors should be considered in relation to career development because emotions are closely related to work experiences and career-related choices (Kidd, 1998, 2004). Also, in a precarious and insecure labor market, uncertainty may lead to excessive anxiety (Laugesen et al., 2003), depression (Dugas et al., 2004), and avoidance (Buhr and Dugas, 2002). Thus, regulating such negative emotions becomes important in the rapidly changing world. Emotion regulation indeed is related to employability (Panari et al., 2020). Thus, investigating emotion regulation as adaptivity is important concerning career-related variables such as CDMSE, the adaptive response of the current study.

However, previous studies on CDMSE have mostly focused on the role of emotional intelligence which included emotion regulation as one of its sub-factors. Moreover, the results regarding the relationship between emotion regulation, as a sub-factor of emotional intelligence, and CDMSE were inconsistent. That is, regulation of emotions did not predict CDMSE in Santos et al.’s (2018) study but had a positive association with a medium effect size in the studies by Brown et al. (2003) and Jiang (2014). Since there are limited studies examining emotion regulation as a separate factor for CDMSE, it would be necessary to examine the relationship between the two variables to provide additional evidence.

While emotion regulation is a complex process involving biological, social, behavioral, and cognitive elements, Garnefski et al. (2001) developed the Cognitive Emotion Regulation Questionnaire (CERQ) to resolve any conceptual confusion by focusing on cognitive measures that are taken to regulate emotions. Garnefski et al. (2001) theoretically divided CER into more adaptive and less adaptive strategies and postulated that individuals using more adaptive strategies report fewer symptoms of depression and anxiety while those using less adaptive strategies report more symptoms of depression and anxiety. In this study, these two categories are referred to as adaptive CER and maladaptive CER. Studies examining CER with career-related variables are scarce, but previous studies have often examined adaptive CER and maladaptive CER separately. For instance, adaptive CER was found to be negatively associated with employment stress while maladaptive CER had a positive correlation with employment stress (Lee and Kim, 2012). In a study by Vanderhasselt et al. (2014), adaptive CER was found to weaken the relationship between dysfunctional attitudes and the depressive symptoms of undergraduate students in stressful situations while maladaptive CER had a direct positive relationship with dysfunctional attitudes and depressive feelings. Based on the theoretical and empirical evidence, the following hypotheses are proposed:

Hypothesis 1: Adaptive cognitive emotion regulation is positively related to CDMSE.

Hypothesis 2: Maladaptive cognitive emotion regulation is negatively related to CDMSE.

### Cognitive emotion regulation and career adaptability

According to the career construction model of adaptation, CA refers to coping resources that are predicted by stable personality traits or individual differences such as cognitive flexibility and willingness to adapt (Savickas and Porfeli, 2012; Savickas, 2013; Rudolph et al., 2017). Based on the model, CER may be a predictor of CA because CER is an individual’s cognitive coping strategy, and specific coping is closely related to personality traits (Vollrath and Torgersen, 2000; Connor-Smith and Flachsbart, 2007).

Studies that examine the relationship between emotion regulation and CA seem scarce. Most of the studies have investigated the relationship between CA and emotional intelligence, with emotion regulation as a sub-factor, and have found that emotional intelligence positively predicts CA (Coetzee and Harry, 2014; Parmentier et al., 2019). It has also been found that high CA is closely associated with less negative affect (Fiori et al., 2015). Based on these studies, the following hypotheses are proposed:

Hypothesis 3: Adaptive cognitive emotion regulation is positively related to career adaptability.

Hypothesis 4: Maladaptive cognitive emotion regulation is negatively related to career adaptability.

### Career adaptability and career decision-making self-efficacy

Career decision-making self-efficacy and CA have been examined as relating variables in numerous studies. In most of the previous studies, CDMSE was examined as a mediating variable leading to CA. For instance, Pambudi et al. (2019) found the mediating effect of CDMSE between the effect of a psychoeducational group and CA, and Hou et al. (2014) found the mediating role of CDMSE between proactive personality and CA. Hamzah et al. (2021) found a strong linear relationship between CDMSE and CA as well as a mediating effect of CDMSE between emotional intelligence, self-esteem, and CA.

However, the career construction model of adaptation illustrates the mechanism by which CA operates in the process of adaptation (Savickas and Porfeli, 2012; Savickas, 2013; Hirschi et al., 2015; Rudolph et al., 2017; Johnston, 2018). That is, CA refers to adaptive resources which lead to adaptive behaviors or responses that help to accomplish developmental tasks in the changing context (Savickas and Porfeli, 2012; Savickas, 2013). CDMSE has been identified as one of the adaptive responses resulting from CA (Rudolph et al., 2017; Johnston, 2018). Thus, to find supporting evidence for the career construction model of adaptation, the following hypothesis is generated:

Hypothesis 5: Career adaptability is positively related to CDMSE.

### The mediating role of career adaptability

Various studies investigated the mediating role of CA in relation to other variables in the framework of the career construction model of adaptation. Nilforooshan and Salimi (2016) found the mediating effect of CA between personality and career engagement. In Nilforooshan’s (2020) study which conducted a multiple mediation model to test the career construction model of adaptation, CA was found to play the mediating role between future work self and proactivity as adaptivity and career decision self-efficacy and career engagement as adaptive responses. Öztemel and Akyol (2021) also found that CA had a mediating effect on self-esteem and career construction behavior.

In the Korean context, Shin and Lee (2018) examined the relationship between regulatory focus, CA, and CDMSE following the career construction model of adaptation and found the mediating role of CA between the other two variables. Kim (2022) also conducted a study with office workers to confirm the adaptation model and found the mediating effect of CA between proactivity and job crafting. To contribute to the research, the current study also adheres to the model of adaptation, proposing that CER predicts CA which in turn predicts CDMSE. Hence, the study examines the following hypotheses:

Hypothesis 6: Career adaptability mediates the relationship between adaptive cognitive emotion regulation and CDMSE.

Hypothesis 7: Career adaptability mediates the relationship between maladaptive cognitive emotion regulation and CDMSE.

## Materials and methods

### Participants

Initially, a total of 361 undergraduate students facing a school-to-work transition in Korea voluntarily consented to participate in this study. Before collecting data, the ethical considerations of the study were evaluated and approved by the Institutional Review Board of Sejong University, South Korea (SJU-2020-004 approved on December 2, 2020). Data were collected in December 2020 by sending an online survey link to university students who were enrolled in a nationwide panel of a data collecting institute. The participants had been informed of the purpose of the study, possible beneficial and harmful outcomes of participation, and that they could stop participating in the study at any time without any disadvantage. Using a screening question, only the students in the 3rd and 4th year in a 4-year university were included because they are generally more actively involved in job-search-related activities in South Korea compared to students in the 1st and 2nd year in university. It took approximately 15 min for the participants to complete the online survey. The gender composition of the participants was 54.8% female and 45.2% male, and the majority of the participants were in their 4th year in university (65.4%). The majors of the participants were liberal arts and social sciences (31.6%), natural sciences and engineering (29.1%), economics and business (16.6%), medicine and pharmacology (10.5%), arts and kinesiology (7.2%), and undefined (1.1%). The mean age of the participants was 24.08 (*SD* = 1.23), and 75.9% of them responded that they were actively seeking a job at the time of participating in the survey. Since four participants did not provide their majors, they were excluded from analysis. The resulting number of participants included in the final analysis was 357, and all participants responded to all survey questions without any missing information.

### Measurement instruments

#### Cognitive emotion regulation

The Cognitive Emotion Regulation Questionnaire (CERQ), originally developed by Garnefski et al. (2001) and validated in Korea by Ahn et al. (2013), was used to measure CER. It consists of 35 items measured by a 5-point Likert scale (1: almost never; 5: almost always). However, we used only 34 items in the current study since the excluded item generally had a very weak relationship with the other items. CERQ includes nine subscales, among which five are more adaptive regulations, namely positive refocusing, refocus on planning, positive reappraisal, putting into perspective, and acceptance, with sample items such as “I think of nicer things than what I have experienced” and “I think that I must learn to live with it.” Subscales of maladaptive regulations include rumination, self-blame, blaming others, and catastrophizing with sample items such as “I feel that I am the one to blame for it” and “I dwell upon the feelings the situation has evoked in me.” The Cronbach’s αs for the nine subscales ranged from 0.68 to 0.83 in the study by Garnefski et al. (2001), and from 0.63 to 0.89 in the study by Ahn et al. (2013). In the present study, the total-score Cronbach’s αs were 0.89 for adaptive CER (18 items) and 0.87 for maladaptive CER (16 items). The sub-scale Cronbach’s αs for adaptive CER ranged from 0.65 to 0.83 while those for maladaptive CER ranged from 0.70 to 0.84.

#### Career adaptability

Career adaptability was measured using the Career Adapt-Ability Scale (CAAS) originally developed by Savickas and Porfeli (2012) and translated into Korean by Tak (2012). In Jeong’s (2013) study, the researcher used Tak’s (2012) Korean translation of CAAS and took several steps to refine some of the expressions to reduce the possibility of misinterpretation and ambiguity. The current study used Jeong’s (2013) refined version which consists of 24 items measured by a 5-point Likert scale (1: not strong; 5: strongest). There are four subscales, concern (e.g., “preparing for the future”), control (e.g., “counting on myself), curiosity (e.g., “exploring my surroundings”), and confidence (e.g., “performing tasks efficiently”), and a higher total score means higher CA. The Cronbach’s αs of the four subscales ranged from 0.80 to 0.93 in Tak (2012) and from 0.71 to 0.90 in Jeong and Jyung (2015). The total-score Cronbach’s α in the present study was 0.95 while the sub-scale Cronbach’s αs ranged from 0.83 to 0.88.

#### Career decision-making self-efficacy

Betz et al. (1996) developed the short form of the Career Decision-Making Self-Efficacy Scale (CDMSES-SF) which includes 25 items and five sub-constructs (accurate self-appraisal, gathering occupational information, goal selection, making plans for the future, and problem-solving). Lee and Lee (2000) translated the CDMSES-SF into Korean, using a 5-point Likert scale to measure each item (1: no confidence at all, 5: complete confidence). Lee and Lee (2000) reported the internal consistency reliability of the subscales ranging from 0.70 to 0.79 but did not report the construct validity. While Lee et al. (2007) reported inconsistent factor structure (three-correlated factor model) with the original study (Betz et al., 1996), the evidence for the construct validity of the CDMSES-SF is scant for Korean university students. In the current study, items that were relevant to the sample and research questions were selected from Lee and Lee’s (2000) version (e.g., “Decide what you value most in an occupation” and “Choose a career that will fit your interest”). The items on gathering occupational information (e.g., through the library) were excluded because they do not reflect the current use of online resources; and the items specific to making decisions and changes about university majors were also eliminated because the current study focused on decision making about job opportunities. We included 13 items to measure CDMSE comprising four subscales: goal selection, making plans for the future, problem-solving, and accurate self-appraisal. The higher total score indicated higher CDMSE. The total-score Cronbach’s α for CDMSE in the present study was 0.87 while the sub-scale Cronbach’s αs ranged from 0.68 to 0.72.

#### Control variables

Based on previous studies that examined university students’ career-related variables, we controlled for some of the demographic factors, such as gender, major, and job-searching status for the final outcome (CDMSE) of the model. First, we included gender as a control variable because previous studies reported an inconsistent relationship between gender and the level of CDMSE (e.g., Chung, 2002; Chen et al., 2021). Second, we controlled for students’ majors because previous studies have found that majors, namely science/engineering and humanities/social science were associated with different levels of career-related variables, such as career decision (Kim and Ra, 2020), career adaptabilities (Shim and Lee, 2015), and job-seeking stress (Kang and Jung, 2021). Finally, we added job-searching status as one of the control variables. Although no studies have directly investigated the relationship between job-searching status and CDMSE to our knowledge, job-seeking stress is negatively correlated with CDMSE in the Korean context (Kim and Lee, 2015; Chae, 2019). For the selected control variables, the reference categories were “female” for gender (female = 0 vs. male = 1), “not searching” for job-searching status (not searching = 0 vs. searching = 1), and “natural sciences and engineering” for major (natural sciences/engineering = 00 vs. humanities/social sciences = 10 vs. arts/kinesiology = 01), respectively.

### Analytical procedure

In the screening phase, *jamovi 2.5.5* was used to examine the descriptive statistics of the data to inspect possible outliers. Then, we examined the bivariate correlations among the studied variables: adaptive CER, maladaptive CER, CA, and CDMSE. We also conducted Harman’s (1976) single factor test to detect the common method bias. Before choosing an estimation method for confirmatory factor analysis (CFA) and path analysis, Mardia’s test was conducted to examine multivariate normality using the QunatPsy package of R. The ensuing major analysis phase consisted of psychometric analysis and path analysis which were conducted using M*Plus*8.

#### Harman’s single factor test

By the nature of the study design (i.e., cross-sectional survey research with self-reported questionnaires) the relationship among the studied variables might be inflated or deflated due to common method variance (CMV) (Podsakoff et al., 2003, 2012). To detect CMV, we conducted Harman’s single factor test for the 71 items of adaptive and maladaptive CER, CA, and CDMSE using exploratory factor analysis with principal axis extraction while fixing the number of factors to one. The chosen program was *jamovi 2.5.5* for Harman’s single factor test.

#### Psychometric analysis

Confirmatory factor analysis was conducted to examine the factor structure of the four variables: adaptive CER, maladaptive CER, CA, and CDMSE. First, we tested the five-correlated factor (positive refocusing, refocus on planning, positive reappraisal, putting into perspective, and acceptance) model with 18 items for adaptive CER and the second-order factor model with the five factors as indicators for higher-order factor (i.e., adaptive CER). Second, we tested the four-correlated factor (rumination, self-blame, blaming others, and catastrophizing) model with 16 items for maladaptive CER while investigating the adequacy of the second-order factor model posing a general factor for the four sub-factors. Third, we tested the four-correlated factor (concern, control, curiosity, and confidence) model with 24 items for CA while testing the second-order factor model with the four factors as indicators. Finally, we tested the four-correlated factor (goal selection, making plans for the future, problem-solving, and accurate self-appraisal) model with the 13 items for CDMSE and the second-order factor model with the four factors governed by a general factor, namely CDMSE. The appropriateness of a CFA model was evaluated based on the alternative fit indices, such as the root mean square of approximation (*RMSEA*), the comparative fit index (*CFI*), and the standardized root mean squared residual (*SRMR*) rather than the chi-square (χ*^2^*) fit statistic due to its poor performance with a large sample (Brown, 2015), particularly, combined with model complexity (Kenny and McCoach, 2003). The following criteria for an acceptable CFA model were employed: *RMSEA* ≤ 0.08, *CFI* ≥ 0.90, and *SRMR* ≤ 0.08 (Barrett, 2007; Brown, 2015). *M*Plus 8.0 was used to conduct CFA. Then, we examined the internal consistency reliability of each scale at both whole-scale and sub-scale levels using *jamovi 2.5.5*.

#### Path analysis

**FIGURE 1:**
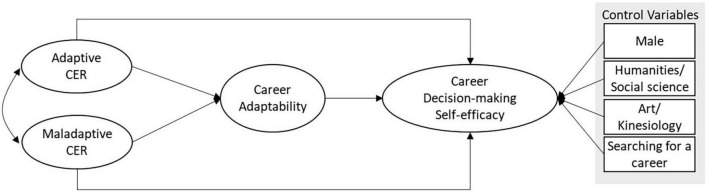
Path analysis model among cognitive emotion regulation, career adaptability, and career decision-making self-efficacy with control variables (gender, major, and job-searching status).

As presented in Figure 1 we tested the path analysis model in which both adaptive CER and maladaptive CER predict CA, and in turn, CA predicts CDMSE. CDMSE was also predicted by adaptive and maladaptive CER while controlled for gender, major, and job-searching status. In the tested model, adaptive CER and maladaptive CER are assumed to be correlated to each other. The same criteria were used to assess the adequacy of the path analysis model as in the CFA: *RMSEA* ≤ 0.08, *CFI* ≥ 0.90, and *SRMR* ≤ 0.08 (Barrett, 2007; Brown, 2015). Then, we evaluated the mediation effects of CA between either adaptive CER or maladaptive CER and CDMSE using bootstrap confidence interval according to Preacher and Hayes (2004).

## Results

### Descriptive statistics and bivariate correlations

We inspected the data based on the descriptive statistics and found that no outliers existed. The descriptive statistics of the main variables (composite scores made by averaging all item scores under each variable) are displayed in Table 1. The mean scores of adaptive CER, maladaptive CER, CA, and CDMSE were 3.48 (*SD* = 0.54), 2.73 (*SD* = 0.63), 3.76 (*SD* = 0.58), and 3.46 (*SD* = 0.55), respectively. The level of maladaptive CER was slightly lower than adaptive CER, which means that the participants used adaptive CER more than maladaptive. The skewness of the variables ranged from –0.09 to –0.70, indicating all variables were somewhat negatively skewed, but all values did not exceed the acceptable range (<| 2|) for retaining the univariate normality assumption (George, 2011). The kurtosis values of the variables ranged from –0.03 to 2.02, indicating all variables were slightly leptokurtic except for maladaptive CER, while all values were less than 2 or very close to 2 which is within the range for univariate normality based on George (2011). However, as univariate normality was not a sufficient condition for multivariate normality, we conducted Mardia’s test as well. The multivariate kurtosis of adaptive CER, maladaptive CER, CA, and CDMSE was 45.93 (*p* < 0.001), indicating that multivariate normality was rejected.

**TABLE 1:** Descriptive statistics and bivariate correlations of the main variables.

Variable	*Mean*	*SD*	*Skew.*	*Kurt.*	*Spearman’ correlation*			
					1	2	3	4
(1) Adaptive CER	3.48	0.54	–0.60	2.02	–	–	–	–
(2) Maladaptive CER	2.73	0.63	–0.09	–0.03	–0.07^ns^	–	–	–
(3) CA	3.76	0.58	–0.70	1.66	0.58***	–0.20***	–	–
(4) CDMSE	3.46	0.55	–0.50	1.18	0.50***	–0.14**	0.77***	–

SD, standard deviation; Skew., skewness; Kurt., kurtosis; ns, non-significant correlation. ***p < 0.001; **p < 0.01.

Since the multivariate normality was not sustainable for the main variables, we refer to Spearman’s correlation. Table 2 presents information regarding the bivariate correlations among the main variables. First, adaptive CER was positively correlated with CA (*r* = 0.58; *p* < 0.01) and CDMSE (*r* = 0.50; *p* < 0.001). Yet, the correlation between adaptive CER and maladaptive CER was not statistically significant (*r* = –0.07; *p* = 0.196). Although no prior study directly examined the correlation between adaptive CER and maladaptive CER, the study by Balzarotti et al. (2016) reported that most of the sub-factors of maladaptive CER do not have significant correlations with those of adaptive CER. Also, the nine CER strategies can theoretically be classified into adaptive and maladaptive CER (Garnefski et al., 2001), but this does not mean that individuals using more adaptive CER necessarily use less maladaptive CER, and vice versa. Hence, adaptive CER and maladaptive CER are not opposing strategies, but just different strategies associated with more adaptive or maladaptive mental health outcomes, respectively. Second, maladaptive CER was negatively correlated with CA (*r* = –0.17; *p* < 0.001) and CDMSE (*r* = –0.14; *p* = 0.010). Third, the correlation between CA and CDMSE was positive and very high (*r* = 0.77; *p* < 0.001).

**TABLE 2:** Confirmatory factor analysis results.

Measurement	Model	χ*^2^*	*df*	*RMSEA*	*CFI*	*SRMR*
*Adaptive CER*	Five-correlated factor model	210.839***	125	0.044	0.955	0.049
	Second-order factor model	270.142***	130	0.055	0.927	0.061
*Maladaptive CER*	Four-correlated factor model	264.237***	97	0.069	0.915	0.058
	Second-order factor model	275.301***	100	0.070	0.910	0.064
*CA*	Four-correlated factor	547.997***	246	0.059	0.918	0.047
	Second-order factor model	556.294***	248	0.059	0.916	0.047
*CDMSE*	Four-correlated factor	157.148***	59	0.068	0.921	0.052
	Second-order factor model	165.771***	62	0.068	0.917	0.054

***p < 0.001.

### Harman’s single factor test

We conducted Harman’s single factor test to examine the method effect due to the design of the current study (i.e., cross-sectional self-reported survey research). The result of Harman’s single factor test indicated that only 25.4% of the variance of the 71 items was accounted for by the single factor assumed to be the effect of the common method. Hence, we garnered that the problem of CMV was not prominent in our study based on the criterion (>50%) in Podsakoff et al. (2003).

### Psychometric analysis

We conducted a set of confirmatory factor analyses to investigate the construct validity evidence for the measurements. Based on the Mardia’s kurtosis values of the 18 adaptive CER items, 16 maladaptive CER items, 24 CA items, and 13 CDMSE items, which were 474.65 (*p* < 0.001), 368.54 (*p* < 0.001), 930.09 (*p* < 0.001), and 262.03 (*p* < 0.001), respectively, we employed the robust Maximum Likelihood estimation method which was recommended for non-normal data (Brown, 2015).

Table 2 provides the results of the set of CFAs. For adaptive CER, both the five-correlated factor model [χ*^2^*_(_*_*df*_*_= 125)_ = 210.839, *p* < 0.001; *RMSEA* = 0.044; *CFI* = 0.955; *SRMR* = 0.049] and second-order factor model [χ*^2^*_(_*_*df*_*_= 130)_ = 270.142, *p* < 0.001; *RMSEA* = 0.055; *CFI* = 0.927; *SRMR* = 0.061] were acceptable. For maladaptive CER, both the four-correlated factor model [χ*^2^*_(_*_*df*_*_= 97)_ = 264.237, *p* < 0.001; *RMSEA* = 0.069; *CFI* = 0.915; *SRMR* = 0.058] and second-order factor model [χ*^2^*_(_*_*df*_*_= 100)_ = 275.301, *p* < 0.001; *RMSEA* = 0.070; *CFI* = 0.910; *SRMR* = 0.064] were adequate after imposing one correlation between unique factors of 9th and 17th items. This is because the correlation between these two items exhibited the largest modification index, and the items, which belong to the same sub-factor ‘blaming others,’ had similar contents: “I feel that others are responsible for what has happened” and “I feel that basically cause lies with others.” For CA, both the four-correlated factor model [χ*^2^*_(_*_*df*_*_= 246)_ = 547.997, *p* < 0.001; *RMSEA* = 0.059; *CFI* = 0.918; *SRMR* = 0.047] and second-order factor model [χ*^2^*_(_*_*df*_*_= 248)_ = 556.294, *p* < 0.001; *RMSEA* = 0.059; *CFI* = 0.916; *SRMR* = 0.047] reasonably fitted to the data. For CDMSE, both the four-correlated model [χ*^2^*_(_*_*df*_*_= 59)_ = 157.148, *p* < 0.001; *RMSEA* = 0.068; *CFI* = 0.921; *SRMR* = 0.052] and second-order factor model [χ*^2^*_(_*_*df*_*_= 62)_ = 165.771, *p* < 0.001; *RMSEA* = 0.068; *CFI* = 0.917; *SRMR* = 0.054] were appropriate. Since the second-order factor model of every scale was acceptable, using the total score for the path analysis model was empirically legitimate. More detailed information regarding the tested CFA models and standardized parameter estimates can be found in the Supplementary material.

Table 3 provides the Cronbach’s α scores at both whole-scale and sub-scale levels. The whole-scale Cronbach’s αs were 0.89, 0.87, 0.95, and 0.87 while the subscale Cronbach’s αs ranged from 0.65 to 0.83, from 0.70 to 0.84, from 0.83 to 0.88, and from 0.68 to 0.72 for adaptive CER, maladaptive CER, CA, and CDMSE, respectively. All Cronbach’s αs were satisfactory based on the criteria (>0.70) suggested by Nunnally and Bernstein (1994) except for two values, namely, ‘putting into perspective’ of adaptive CER and ‘making plans for the future’ of CDMSE.

**TABLE 3:** Internal consistency reliability coefficients of the scales.

Scale	Cronbach’s α	Scale	Cronbach’s α
*Adaptive CER*	0.89	*CA*	0.95
Positive refocusing	0.83	Concern	0.86
Refocus on planning	0.75	Control	0.86
Positive reappraisal	0.79	Curiosity	0.83
Putting into perspective	0.65	Confidence	0.88
Acceptance	0.71		
		*CDMSE*	0.87
*Maladaptive CER*	0.87	Accurate self-appraisal	0.72
Rumination	0.70	Goal selection	0.71
Self-blame	0.82	Making plans for the future	0.68
Blaming others	0.84	Problem-solving	0.71
Catastrophizing	0.79		

### Path analysis

**FIGURE 2:**
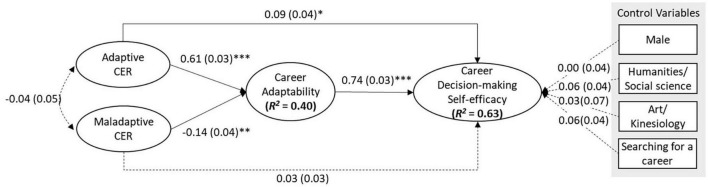
Path analysis results with the standardized path coefficients. ^**^p < 0.01, ^***^p < 0.001; a number in the parenthesis indicate the corresponding standard error for each of the standardized path coefficients; we provided unstandardized path coefficients for control variables since they were either binary or dummy-coded variable; a dotted line indicates a non-significant path.

To test the hypothesized model (Figure 1) of adaptive CER, maladaptive CER, CA, and CDMSE we conducted path analysis using the robust Maximum Likelihood estimator. The tested path analysis model appeared to be adequate based on the model fit indices [χ*^2^*_(_*_*df*_*_= 12)_ = 16.56, *p* = 0.17; *RMSEA* = 0.033; *CFI* = 0.991; *SRMR* = 0.056] as shown in Figure 2. The *R*^2^s of CDMSE and CA were 0.63 and 0.40, respectively, indicating that approximately 63% of the variance of CDMSE was explained by adaptive and maladaptive CER, CA, and control variables (gender, major, and job-search status), and 40% of the variance of CA was explained by adaptive and maladaptive CER.

Both Table 4 and Figure 2 provide the path analysis results with path coefficients of direct effects. The standardized path (β) from adaptive CER to CDMSE was 0.09 (*SE* = 0.04; *p* < 0.05), supporting Hypothesis 1. However, β from maladaptive CER to CDMSE was not significant, indicating that Hypothesis 2 was not supported. β from adaptive CER to CA was 0.61 (*SE* = 0.03; *p* < 0.001), indicating a strong positive relationship, while β from maladaptive CER to CA was –0.14 (*SE* = 0.03; *p* < 0.01), supporting both Hypotheses 3 and 4. Since β from CA to CDMSE was 0.74 (*SE* = 0.03; *p* < 0.001), Hypothesis 5 was supported, which indicates that CA was positively related to CDMSE after controlling for gender, job-searching status, and majors. As presented in Table 4, none of the control variables had a significant effect on CDMSE.

**TABLE 4:** Path analysis results.

Direct effect	*B* (*SE*_*B*_)	β (*SE*_β_)
Adaptive CER → CA	0.66 (0.05)***	0.61 (0.03)***
Maladaptive CER → CA	–0.13 (0.04)**	–0.14 (0.04)**
Adaptive CER → CDMSE	0.09 (0.04)*	0.09 (0.04)*
Maladaptive CER → CDMSE	0.02 (0.03)	0.03 (0.03)
CA → CDMSE	0.69 (0.04)***	0.74 (0.03)***
Male → CDMSE	0.00 (0.04)	–
Humanities/Social Sciences→ CDMSE	0.06 (0.04)	–
Art/Kinesiology → CDMSE	0.03 (0.07)	–
Searching for a career → CDMSE	0.06 (0.04)	–

B, unstandardized path coefficient; SE_B_, standard error of B; β, standardized path coefficient; SE_β_, standard error of β. ***p < 0.001; **p < 0.01; *p < 0.05.

We used a 95% bias-correct bootstrapping confidence interval with 2000 bootstrapping samples to test the mediation effect of CA between adaptive CER and CDMSE and that between maladaptive CER and CDMSE. Table 5 presents the path coefficients of the indirect effects and their bootstrap confidence intervals (BS-CIs). The standardized indirect effect of adaptive CER through CA to CDMSE was 0.45 (*SE* = 0.05; *p* < 0.001) with a 95% BS-CI of [0.364, 0.543], which supported Hypothesis 6 that CA would mediate the relationship between adaptive CER and CDMSE. Since the direct effect from adaptive CER to CDMSE was statistically significant, we can consider that CA partially mediated the relationship between adaptive CER and CDMSE, and that the total effect of adaptive CER to CDMSE was positive and significant (β = 0.536; *p* < 0.001). The standardized indirect effect of maladaptive CER through CA to CDMSE was –0.11 (*SE* = 0.03; *p* < 0.01) with a 95% BS-CI of [–0.18, –0.05], indicating that Hypothesis 7 was supported. The non-significant direct effect from maladaptive CER on CDMSE combined with the significant bivariate correlation between them allowed us to interpret that CA fully mediated the relationship between maladaptive CER and CDMSE. However, the total effect of maladaptive CER to CDMSE was not significant (β = –0.08; *p* = 0.070), which means that the overall effect of maladaptive CER to CDMSE was offset.

**TABLE 5:** Path coefficients and bootstrapping confidence intervals of indirect effects.

Indirect effect	*B* (*SE*_*B*_)	β (*SE_β_*)	BS-CI of β
Adaptive CER → CA → CDMSE	0.46 (0.06)***	0.45 (0.05)***	[0.35, 0.54]
Maladaptive CER → CA → CDMSE	–0.09 (0.03)**	–0.11 (0.03)**	[–0.18, –0.05]

***p < 0.001; **p < 0.01.

## Discussion and conclusion

### Findings and implications

The current study found supporting evidence for the adequacy of the career construction model of adaptation (Savickas, 2013) with adaptive and maladaptive cognitive emotion regulation (CER), CA, and CDMSE in a sample of Korean university students. The findings supported the mediating role of CA between adaptive and maladaptive CER and CDMSE. Specifically, CA partially mediated the effect of adaptive CER on CDMSE. That is, the significant relationship between adaptive CER and CDMSE remained after CA was placed in the model as a mediator. Meanwhile, CA fully mediated the effect of maladaptive CER on CDMSE, since the significant relationship between maladaptive CER and CDMSE disappeared when CA was added to the model as a mediator. The specific findings are as follows.

#### Relationship found between cognitive emotion regulation and career decision-making self-efficacy

Regarding the relationship between CER and CDMSE, adaptive CER was positively associated with CDMSE, supporting Hypothesis 1, although the magnitude of the relationship was not large. The role of emotion in the process of career decision-making has been addressed by many studies, most of which have focused on emotional intelligence in relation to career decision-making-related variables. Specifically, studies have found that emotional intelligence had a positive effect on CDMSE (Jiang, 2016; Song and Shin, 2016; Santos et al., 2018; Park et al., 2019). However, studies examining the relationship between emotion regulation in particular and CDMSE are scarce. In Jiang’s (2014) study, for instance, which verified the positive effect of emotional intelligence on CDMSE, regulation of emotion was one of the sub-factors of emotional intelligence that showed a medium effect size on CDMSE. As examined by Peña-Sarrionandia et al. (2015), emotion regulation and emotional intelligence are independent research traditions that try to explain emotion management; emotion regulation focuses on the process by which one manages emotions, while emotional intelligence focuses on individual differences in emotion management. Thus, the result of the current study is significant in that it provided additional evidence showing that regulating emotions using adaptive cognitive strategies is positively related to CDMSE. This finding is also analogous to the studies supporting a positive relationship between emotion regulation and self-efficacy in an academic domain (Yu, 2013; Yang and Lee, 2020) and between academic emotion regulation and CDMSE (Kim and Ryu, 2017). Han and Moon (2015) also emphasized the role of emotion regulation on CDMSE in the process of career decision-making, and the current study supported the positive role of adaptive CER on CDMSE.

On the other hand, the results showed that maladaptive CER had no significant relationship with CDMSE, which did not support Hypothesis 2. The significant relationship between maladaptive CER and CDMSE disappeared after CA was taken into consideration. It seemed that the strong positive correlation between CA and CDMSE (*r* = 0.77) offset the negative and small correlation between maladaptive CER and CDMSE (*r* = –0.14). That is, CA fully mediated the relationship between maladaptive CER and CDMSE, and more discussions will ensue.

#### Relationship found between cognitive emotion regulation and career adaptability

Adaptive CER was positively correlated with CA, while maladaptive CER was negatively correlated with CA, supporting Hypothesis 3 and 4 respectively. As mentioned previously, CA has more often been examined in relation to emotional intelligence. Since effective regulation of emotions can be encompassed in emotional intelligence (Salovey and Mayer, 1990), the result of the present study is compatible with the previous research by Parmentier et al. (2019) which found the causal relationship between emotional intelligence and CA. Çizel (2018), who examined gender and emotional intelligence as predictors of CA, found that women with higher emotional intelligence had higher CA. In Coetzee and Harry’s (2014) study, CA was predicted by emotional intelligence and also influenced by how one managed one’s own emotions. These previous studies illustrate the effect of emotion and emotional management on CA, and the current study provides additional supporting evidence by verifying the positive relationship between adaptive CER and CA.

Moreover, the current study found a negative relationship between maladaptive CER and CA. Maladaptive CER has been examined in relation to emotional problems (Kraaij et al., 2003; Garnefski and Kraaij, 2006), negative emotions such as depression, anxiety, stress, and anger (Martin and Dahlen, 2005), psychopathology (Garnefski et al., 2017), and problematic behaviors such as excessive smartphone and social media use (Zsido et al., 2021), but there seem to be no studies related to career development. Considering that unpredictable career trajectories may cause negative emotions such as stress and anxiety, it is important to understand how regulating such emotions influences the adaptability of those in their school-to-work transition. The result of the current study, indicating how using maladaptive cognitive strategies to regulate emotions is negatively related to CA, offers the basis for further discussions on the relationship between CER and CA as it relates to the workplace.

#### Relationship found between career adaptability and career decision-making self-efficacy

Career adaptability had a positive relationship with CDMSE, which supports Hypothesis 5. Douglass and Duffy (2015) used the CAAS and found that the total score of CA as well as the scores of the sub-factors had a significant correlation with CDMSE. Duffy et al. (2015) also examined the total score of CA and the scores of the sub-factors and found a significant correlation with CDMSE. However, Stead et al. (2021) conducted a meta-analysis to analyze the relationship between CA and CDMSE and found that estimated correlations between the sub-factors of CA and CDMSE were low to moderate while the total score of CA had a stronger relationship with CDMSE. The result of the current study, which examined the total score of CA with CDMSE and found significant positive relationship, provides support for this previous finding. This finding is also consistent with Nilforooshan (2020)’s study that used a multiple mediation model to investigate the career construction model of adaptation and found a significant positive relationship between CA and CDMSE.

#### Relationships found among cognitive emotion regulation, career adaptability, and career decision-making self-efficacy

Concerning the mediating role of CA between CER and CDMSE (Hypotheses 6), we found that CA partially mediated the relationship between adaptive CER and CDMSE. This result is comparable to various studies that have investigated the mediating role of CA. For instance, Nilforooshan and Salimi (2016) found that CA mediated the relationship between personality and career engagement, and Celik and Storme (2018) found that CA mediated the relationship between trait emotional intelligence and academic satisfaction. In Woo’s (2018) study, CA mediated the relationship between personality traits and intrapreneurship, and in Shin and Lee’s (2018) study, a mediation effect of CA between regulatory focus and CDMSE was found. In alignment with these studies, the present study adds support for the mediating role of CA on the relationship between adaptive CER and CDMSE.

Meanwhile, CA had a mediating effect between maladaptive CER and CDMSE, as postulated in Hypothesis 7. As a whole, however, the effect of maladaptive CER to CDMSE was suppressed based on the non-significant total effect. According to Zhao et al. (2010) the full mediation effect of CA could be interpreted as the complementary mediation effect since the direction of the relationship between maladaptive CER and CDMSE was opposite from the direction between CA and CDMSE. To our knowledge, no study has examined the mediating role of CA on maladaptive CER and CDMSE, and thus, it is not possible to directly compare our results to that of other studies. Instead, we presume that the effect of maladaptive CER on CDMSE can be lessened by CA. For example, university students who face reduced job opportunities after the outbreak of the COVID-19 pandemic might exhibit maladaptive emotion regulation such as self-blame or catastrophizing which may be associated with their lower level of CDMSE. However, university students with high CA might exhibit higher level of CDMSE while being minimally susceptible to the negative effect of maladaptive CER. Of course, it should be noted that our study design does not allow us to interpret the relationship among the three variables as being causal. Therefore, the mediating role of CA between maladaptive CER and CDMSE should be investigated based on an experimental study in the future.

#### Non-significant relationship found between control variables and career decision-making self-efficacy

Students’ gender, major, and job-searching status were selected as control variables for CDMSE, but none of the chosen control variables had a significant relationship with CDMSE. Most of all, gender showed no significant relationship with CDMSE. Previous studies showed inconsistent results. Specifically, Chen et al.’s (2021) study found that male Chinese high school students scored slightly higher on CDMSE than their female counterparts, but Chung’s (2002) study showed that female and male undergraudate students in the USA did not display differences in CDMSE scores. The finding of the current study supports that there are no gender differences regarding the level of CDMSE. CDMSE measured in this study consists of selecting goals, making plans for the future, solving problems, and accurately appraising oneself. These are general career-related tasks, rather than tasks targeting specific jobs which may be perceived as having barriers or being gender biased. Hence, the level of CDMSE may have individual differences but not gender differences.

Moreover, students’ major and job-searching status had no significant relationship with CDMSE. Although no prior study seemed to have directly investigated the effects of major and job-searching status on CDMSE, we added these control variables because major was related to career decision (Kim and Ra, 2020), and job-seeking stress was associated with CDMSE (Kim and Lee, 2015; Chae, 2019). The pandemic situation was also taken into account. The current study was conducted amidst the pandemic during which social distancing and quarantine measures affected fields of work differently. For instance, travel agencies, airlines, and hotels faced big losses and were forced to downsize, while IT industry bloomed as businesses and education switched to non-face-to-face platforms. It seemed arguable that students who were majoring in or searching for a job in fields with different prospects may experience different levels of CDMSE. However, the results showed that the level of CDMSE did not differ based on major and job-searching status. As mentioned earlier, this may be because CDMSE refers to decision-making self-efficacy about general career-related tasks rather than about specific fields of work.

### Significance and implications of the findings

The theoretical and practical implications of the findings of the current study are discussed as follows.

#### Theoretical significance

The findings of this study have theoretical significance in that they provide supporting evidence for Savickas (2013) career construction model of adaptation in the context of Korean university students, addressing the need to investigate the applicability of the model in different cultural contexts (Shin and Lee, 2018). In addition, we incorporated plausible but yet-to-be-tested variables (i.e., adaptive and maladaptive CER) to represent adaptive readiness in the model while confirming the mediating role of CA between adaptive and maladaptive CER and CDMSE. This incorporation of CER provides the groundwork for further discussions on the role of different emotion regulation strategies used in the process of career construction for university students. Studies show that emotion regulation leads to enhanced control and confidence in the process of career construction (Wehrle et al., 2019), and is positively related to career outcomes (Urquijo et al., 2019). However, people regulate their emotions in different ways, some being more adaptive than others. How one regulates negative emotions at times of uncertainty, most recently seen in the effects of the pandemic, can be an important consideration when understanding the process of one’s career construction. Moreover, emotions, and their expressions and regulations, can be understood as social constructs within the sociocultural system (Averill, 1980; Cornelius, 2000), and individuals from different cultural contexts may experience different emotional reactions and use different regulation strategies to adapt to the rapidly changing modern society. Since the current study found the relationship between CER and career-related variables, namely CA and CDMSE, in accordance with the model of adaptation, the results may provide the groundwork based on which the cultural context of emotional reactions to increasingly uncertain environments and the regulation strategies individuals use can be further explored.

#### Practical implications

The results also have practical implications for university counselors and educators working with students in the school-to-work transition in today’s world of rapid advancement and increased uncertainty. First, the impact of emotion regulation on career development should be acknowledged. Counselors and educators should address students’ emotional responses to the ever-changing world of work and examine cognitive strategies that students can use to regulate their emotions. As Garnefski et al. (2002) suggested, assessing all the cognitive emotion regulation strategies together would be effective for a more comprehensive understanding of how the strategies interplay. It should also be noted that the current study was conducted during a surge in the number of individuals infected with COVID-19 in South Korea and that anxiety levels were especially high due to the precariousness across every aspect (e.g., health, education, economy, the job market, etc.) of people’s lives. In particular, COVID-19 struck our economy while yielding an unstable job market, and the university students who were on the verge of the school-to-work transition might have suffered from increased anxiety and uneasiness related to their transition. Although the present study was not designed to capture the direct impact of the pandemic on students’ career construction, it was conducted amidst the pandemic and its findings provide insight into the importance of regulating negative emotions to better assist students in the transition to adapt to the on-going impacts of the pandemic.

Next, interventions are needed for students with maladaptive CER. Previous studies propose that interventions should aim to challenge maladaptive CER and enhance the use of more adaptive CER strategies (Garnefski et al., 2003; Kraaij et al., 2003). More adaptive CER would not only reduce negative emotional responses but also have a positive effect on enhancing CA that is needed to adjust to uncertain situations. Specifically, cognitive therapy approaches such as reframing and cognitive restructuring have been proposed as effective interventions to reduce maladaptive CER and shift to more adaptive strategies (Garnefski and Kraaij, 2007).

Also, considering its mediating effect, the role of CA should be emphasized. CA is malleable and can be gained through training (Koen et al., 2012). Studies have demonstrated that training programs specifically designed to enhance CA have been effective (Koen et al., 2012; Green et al., 2020). In particular, career education programs incorporating meaning-making – one of the key components of career construction counseling (Savickas, 2016) – might help university students further develop their CA. For example, Son (2016) developed a career education program designed to assist Korean university students to engage in meaning-making and found that the program enhanced careers overall. Considering that modern society entails an increasing number of uncontrollable changes, such as the advancement of technology and the pandemic, it becomes more important for individuals to make their own meaning in life, to construct career identities based on a comprehensive exploration of themselves and environments, and to construct their own career trajectory (Savickas, 2016). Therefore, counselors and educators may design training exercises targeting CA as well as meaning-making programs to assist university students in their transition and career construction. Furthermore, since social support is found to be significantly related to CA (Hirschi, 2009; Duffy, 2010; Jung and Cho, 2015), providing counseling services through which counselors and students build a supportive relationship would be an important factor in assisting students.

Since CA indicates psychosocial resources one can draw upon to manage various career-related tasks and challenges, fostering university students’ CA would not only help students make a successful transition into the workforce, but also help them adjust to changes in their workplace. Studies have found that CA is associated with various organizational outcomes, such as career satisfaction (Chan and Mai, 2015), higher employment quality (Koen et al., 2012), and professional wellbeing (Maggiori et al., 2013). CA was also negatively related to turnover intentions (Chan and Mai, 2015). Hence, emphasis on enhancing the role of CA at the university level may be expected to lead to positive organizational outcomes.

Finally, the aforementioned practical implications, namely intervening to assist students in using more adaptive CER and enhancing CA, are related to CDMSE. CDMSE refers to self-efficacy which is not simply related to making decisions about what jobs to select, but to gather the necessary information and making sound decisions for one’s career. Hence, gaining an understanding of CDMSE for university students, who are about to join the world of work, may provide some insight at the organizational level as well. In a study involving professionals in the electronic media industry, CDMSE was found to be positively related to career optimism (Ahmad and Nasir, 2021). In another study with managers in government agencies, it was found that managers with higher CDMSE were more likely to make a turnover decision to stay, and it was suggested that CDMSE helped managers be more proactive in building relationships with supervisors and peers, gain career connectedness, and find work-life balance (Peterson, 2009). Thus, while the current study focused on university students, the relationship between their adaptive readiness, resources and responses found in the study could provide the groundwork for extending the understanding of their adaptation process at the organizational level.

### Limitations and future directions

The present study has several limitations. First, the study was conducted with cross-sectional data to examine the relationships between the variables. To find evidence for causal relationships, longitudinal and experimental studies may be needed. Identifying causal relationships among the variables may provide more rigorous support for the career construction model of adaptation, as well as insight for devising more comprehensive interventions for students.

Second, we tested the CMV using Harman’s single factor test, but the method is known for its insensitivity and inability to handle the CMV. Future studies that use a single-administration self-report survey design should use more robust methods, such as a marker variable (Tehseen et al., 2017), to detect CMV while handling it.

Third, the study used the total scores of the variables to examine their relationships, but to have a more comprehensive understanding of relationships among the examined variables, the sub-factors should be considered in the future. As proposed, interventions to challenge maladaptive CER and enhance CA would be needed to assist university students in their transition to the workforce, and understanding the functions of the variables’ sub-factors would help design specific interventions or training programs.

Fourth, we used only 13 items out of the 25 items in the original CDMSE scale since some item contents did not apply to our study. Therefore, we should be careful to interpret the results related to CDMSE, as they might not be directly comparable to the other studies in which the whole item set was used. Furthermore, in future studies, the reliability and validity of CDMSE should be reevaluated for university students who face a school-to-work transition.

Finally, the generalizability of the results may be limited since the sample of the study consisted only of Korean university students. The circumstances of the labor market and domestic economy, as well as the situations of university students, may look very different in other countries. To extend the generalizability of the results, future study is needed to encompass participants from other cultures and countries.

Despite the limitations, this study contributes to the existing literature by identifying the relationships among adaptive and maladaptive CER, CA, and CDMSE, confirming the career construction model of adaptation. Based on the results of the study, practical interventions can be designed and implemented to assist university students in their transition period in the context of this unpredictable, changing world.

## Data availability statement

The datasets presented in this study can be found in online repositories. The names of the repository/repositories and accession number(s) can be found below: Mendeley Data (doi: 10.17632/cmrrccbmdh.1).

## Ethics statement

The study involving human participants were reviewed and approved by Institutional Review Board of Sejong University. Written informed consent for participation was not required for this study in accordance with the national legislation and the institutional requirements.

## Author contributions

AL and EJ equally contributed to the conceptualization of the study. AL was mainly responsible for writing the introduction, literature review, and discussion sections. EJ took charge of conducting formal data analysis and writing the “Materials and methods” and “Results” sections. Both authors contributed to the article and approved the submitted version.
