# Supplementary material for: University students’ career adaptability as a mediator between cognitive emotion regulation and career decision-making self-efficacy
Source: Front Psychol. 2022 Oct 5;13:896492. doi: 10.3389/fpsyg.2022.896492 (PMC9581253; doi:10.3389/fpsyg.2022.896492)
Supplement: Supplementary file 1 [file Data_Sheet_1.docx]

Supplementary Material


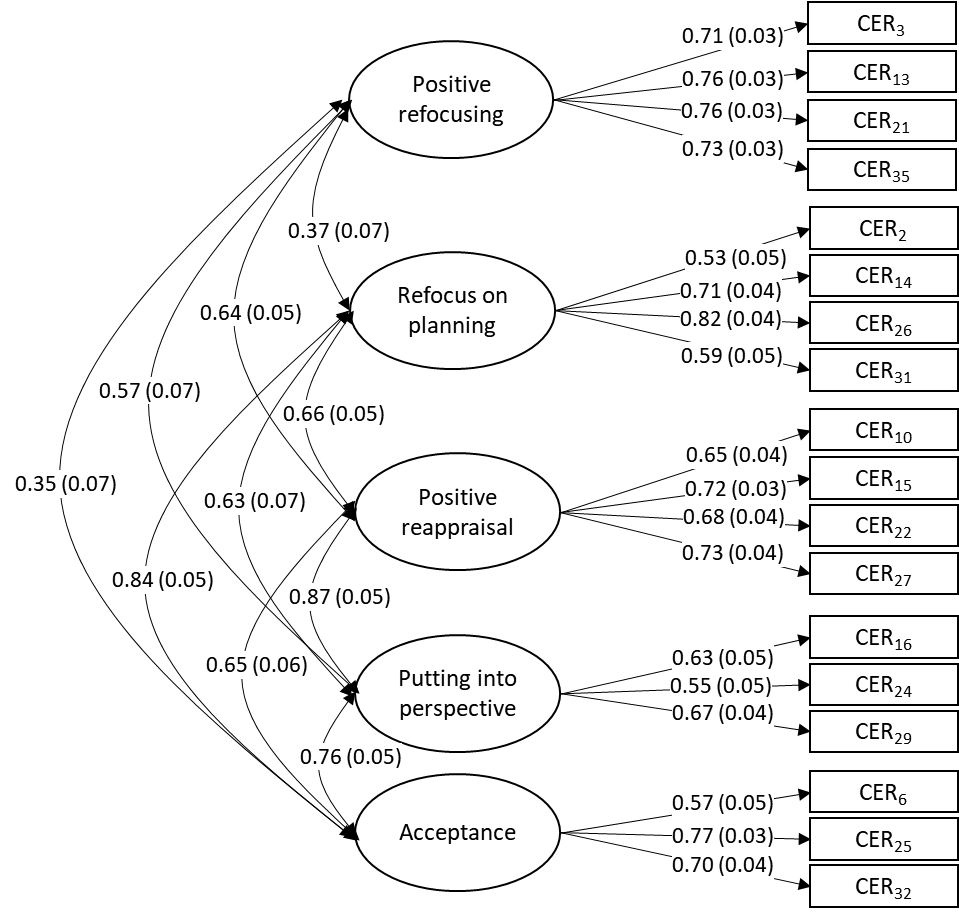


**Supplementary Figure 1.** Five-correlated factor model of adaptive CER with standardized factor loadings and correlations among sub-factors. *Note.* Model fit indices: *χ^2^*_(_*_df_*_=125)_ = 210.839, *p* < 0.001; *RMSEA* = 0.044; *CFI* = 0.955; *SRMR* = 0.049; a value in the parenthesis is standard error of its corresponding parameter estimate.


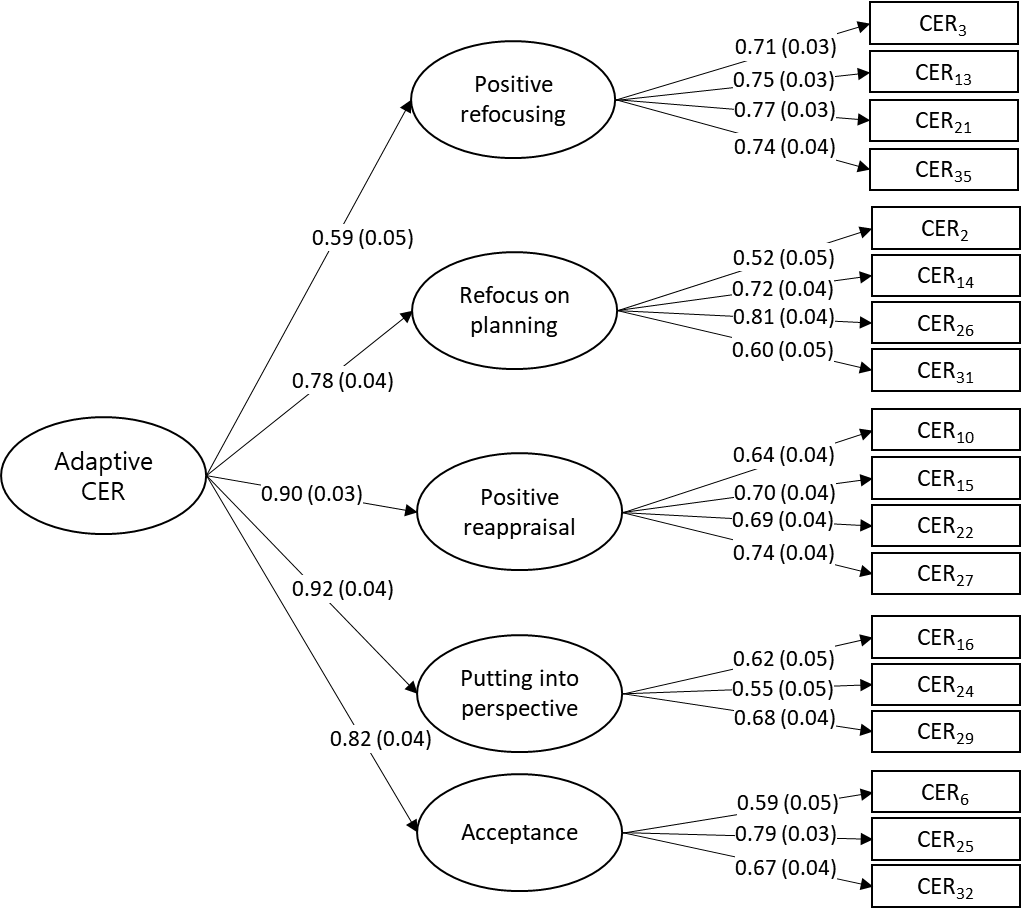


**Supplementary Figure 2.** Second-order factor model of adaptive CER with standardized first- and second-order factor loadings. *Note.* Model fit indices: *χ^2^*_(_*_df_*_=130)_ = 270.142, *p* < 0.001; *RMSEA* = 0.055; *CFI* = 0.927; *SRMR* = 0.061; a value in the parenthesis is standard error of its corresponding parameter estimate.

**
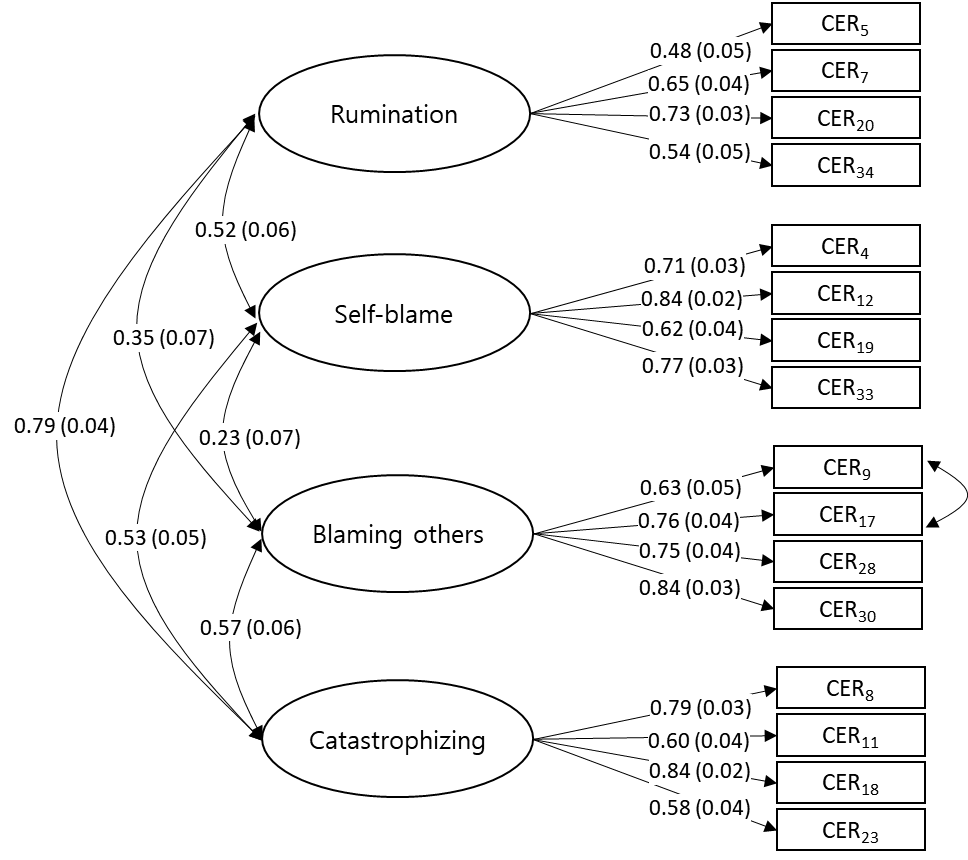
**

**Supplementary Figure 3.** Four-correlated factor model of maladaptive CER with standardized factor loadings and correlations among sub-factors. *Note.* Model fit indices: *χ^2^*_(_*_df_*_=97)_ = 264.237, *p* < 0.001; *RMSEA* = 0.069; *CFI* = 0.915; *SRMR* = 0.058; a value in the parenthesis is standard error of its corresponding parameter estimate; In the model, the correlation between unique factors of CER9 and CER17 was imposed based on the largest modification index from the original model without the correlation.


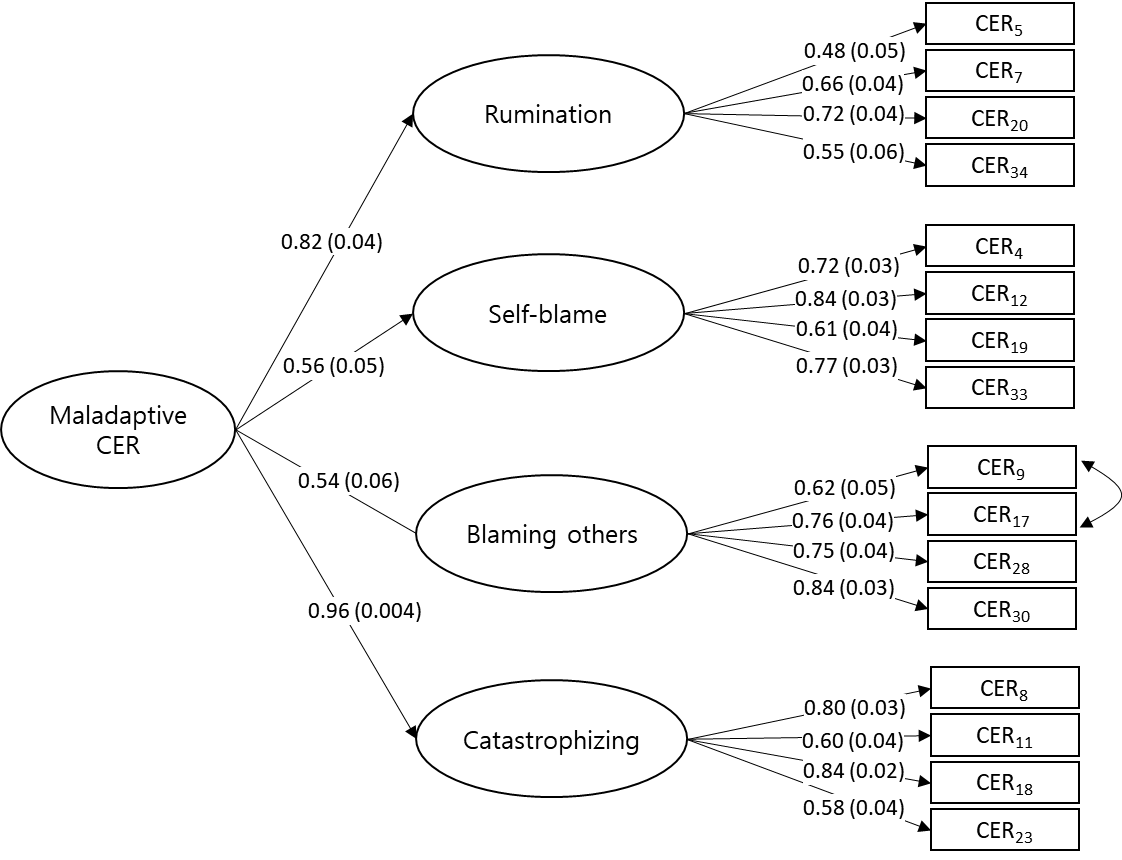


**Supplementary Figure 4.** Second-order factor model of maladaptive CER with standardized first- and second-order factor loadings. *Note.* Model fit indices: *χ^2^*_(_*_df_*_=100)_ = 275.301, *p* < 0.001; *RMSEA* = 0.070; *CFI* = 0.910; *SRMR* = 0.064; a value in the parenthesis is standard error of its corresponding parameter estimate.; In the model, the correlation between unique factors of CER9 and CER17 was imposed based on the largest modification index from the original model without the correlation.


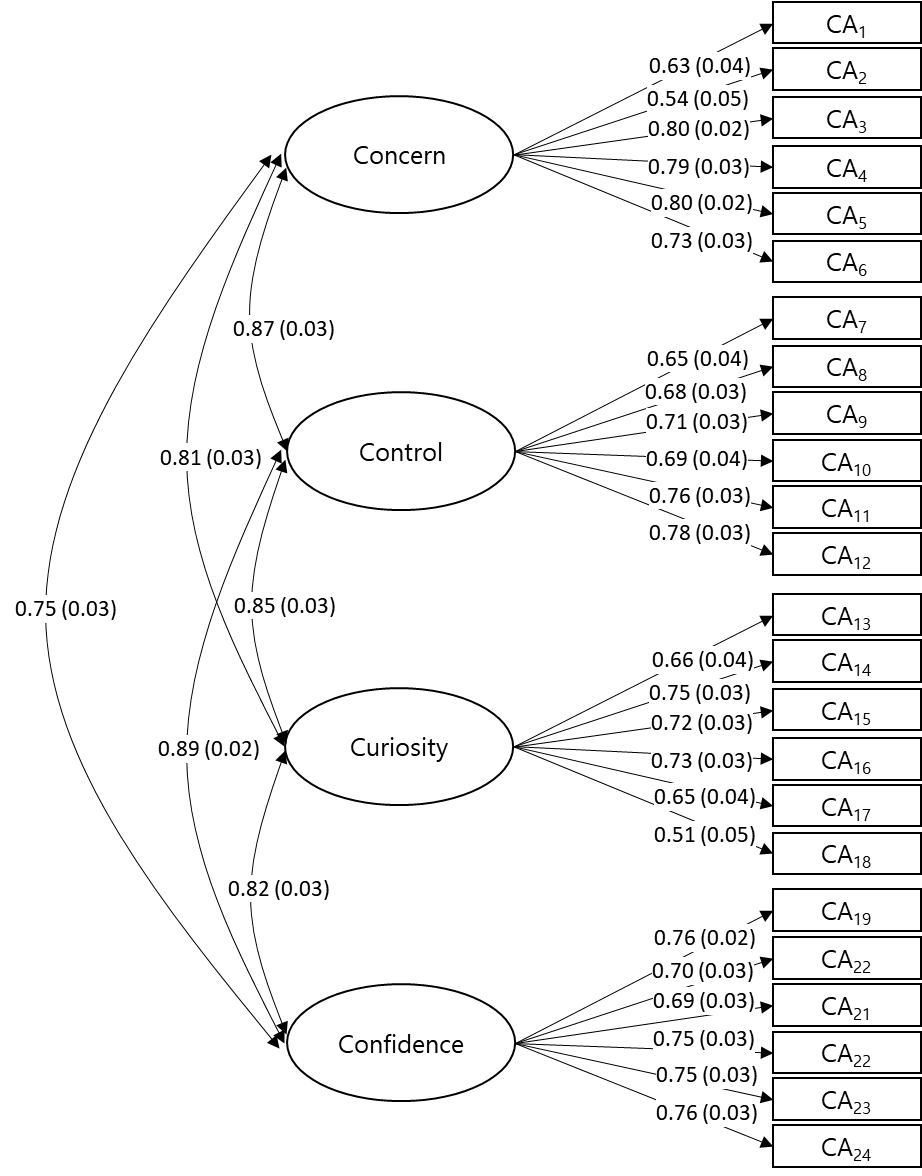


**Supplementary Figure 5.** Four-correlated factor model of CA standardized factor loadings and correlations among sub-factors. *Note.* Model fit indices: *χ^2^*_(_*_df_*_=246)_ = 547.997, *p* < 0.001; *RMSEA* = 0.059; *CFI* = 0.918; *SRMR* = 0.047; a value in the parenthesis is standard error of its corresponding parameter estimate.


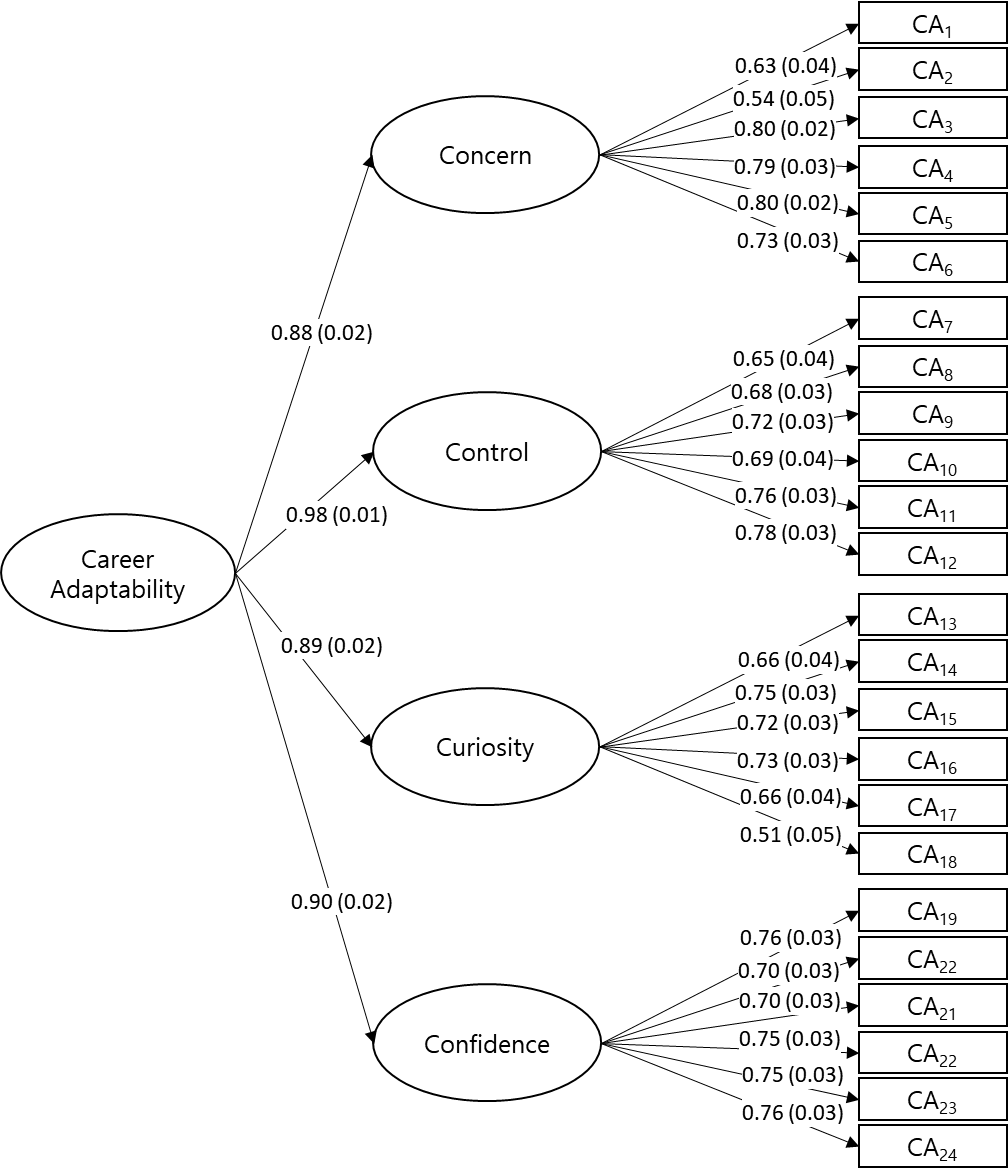


**Supplementary Figure 6.** Second-order factor model of CA with standardized first- and second-order factor loadings. *Note.* Model fit indices: *χ^2^*_(_*_df_*_=248)_ = 556.294, *p* < 0.001; *RMSEA* = 0.059; *CFI* = 0.916; *SRMR* = 0.047; a value in the parenthesis is standard error of its corresponding parameter estimate.


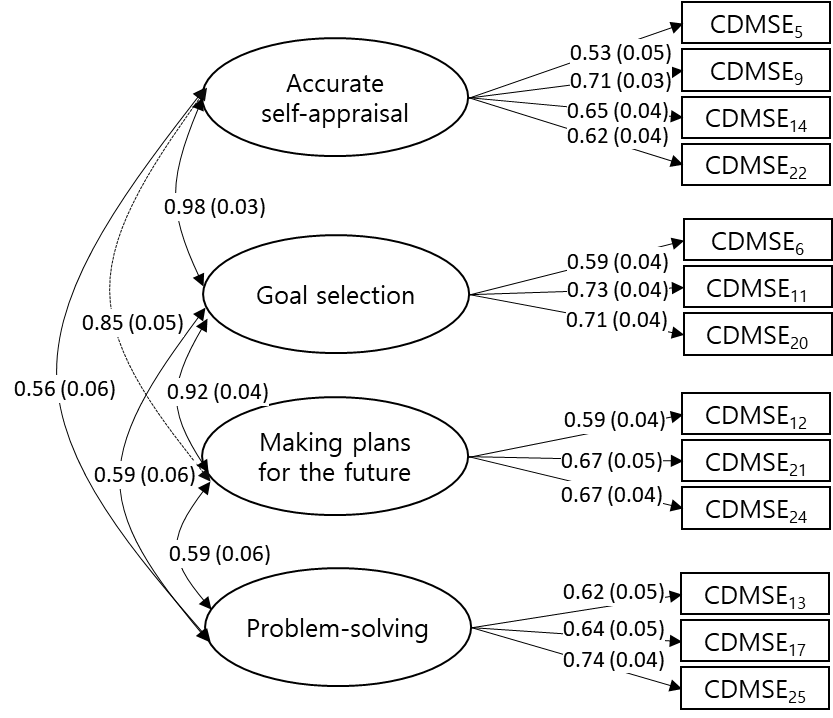


**Supplementary Figure 7.** Four-correlated factor model of CDMSE with standardized factor loadings and correlations among sub-factors. *Note.* Model fit indices: *χ^2^*_(_*_df_*_=59)_ = 157.148, *p* < 0.001; *RMSEA* = 0.068; *CFI* = 0.921; *SRMR* = 0.052; a value in the parenthesis is standard error of its corresponding parameter estimate.


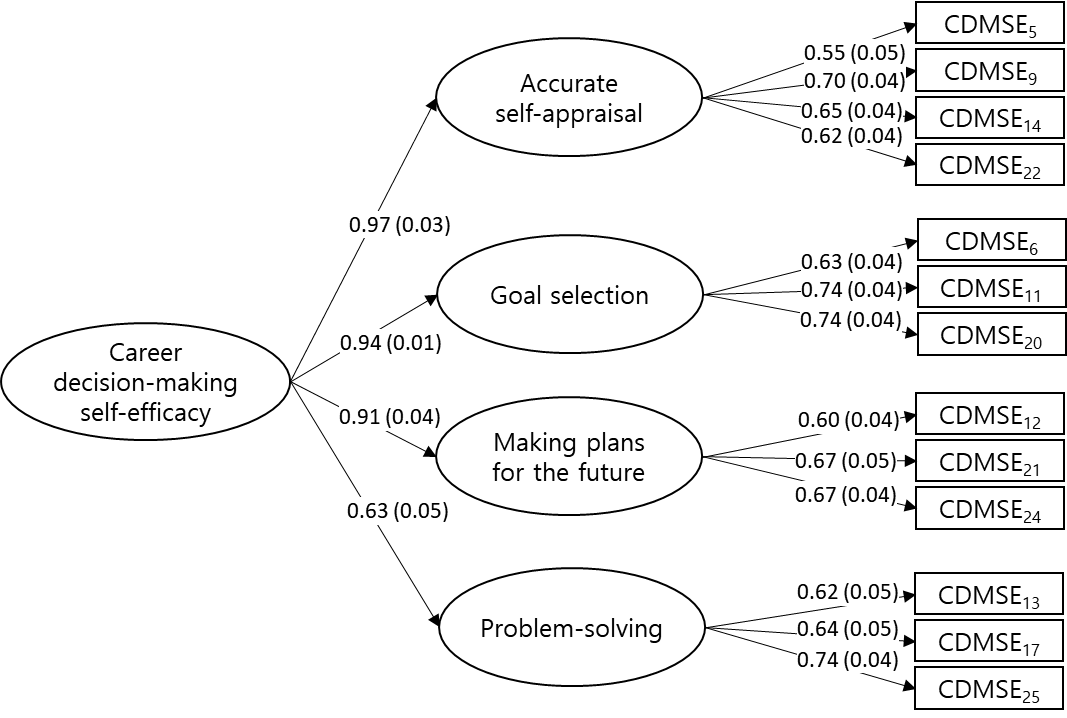


**Supplementary Figure 8.** Second-order factor model of CDMSE with standardized first- and second-order factor loadings. *Note.* Model fit indices *χ^2^*_(_*_df_*_=62)_ = 165.771, *p* < 0.001; *RMSEA* = 0.068; *CFI* = 0.917; *SRMR* = 0.054; a value in the parenthesis is standard error of its corresponding parameter estimate.
